# When Infection Drives Autoimmunity: *Mycobacterium abscessus*–Associated MPO‐ANCA Glomerulonephritis—A Cytotoxic‐Sparing Treatment Approach

**DOI:** 10.1155/crin/7595496

**Published:** 2026-11-15

**Authors:** Muzammeel Hada, Iyad Mansour, Muna Omar, Bijin Thajudeen, Issa Yousef Batarseh, Sanjeeva Weerasinghe, Tahsin Ozpolat

**Affiliations:** ^1^ Department of Nephrology University of Arizona Tucson Arizona USA arizona.edu; ^2^ Department of Medicine University of Arizona Tucson Arizona USA arizona.edu; ^3^ Department of Haematology University of Washington Seattle Washington USA washington.edu

## Abstract

**Background:**

Nontuberculous mycobacterial (NTM) infections are increasingly recognized as triggers of antineutrophil cytoplasmic antibody (ANCA) positivity and secondary ANCA‐associated vasculitis. Differentiating infection‐associated ANCA disease from primary AAV is critical because management and outcomes differ. This case describes an uncommon organism triggering MPO‐ANCA glomerulonephritis and a management approach that avoided cytotoxic agents.

**Case Presentation:**

A 57‐year‐old woman with chronic pulmonary disease presented with acute kidney injury, glomerular‐pattern (dysmorphic) microscopic hematuria, proteinuria, and MPO‐ANCA positivity. Kidney biopsy demonstrated pauci‐immune necrotizing and crescentic glomerulonephritis with marked chronicity (∼70% globally sclerosed glomeruli). Evaluation revealed pulmonary infection with multidrug‐resistant *Mycobacterium abscessus* complex. Given active infection, cyclophosphamide and rituximab were withheld. The patient received pulse intravenous methylprednisolone followed by oral prednisone with targeted antimicrobial therapy.

**Outcome:**

Over approximately 6 months of follow‐up, serum creatinine improved from a peak of 3.9 mg/dL to 2.2 mg/dL and subsequently to 1.9 mg/dL, ESR and CRP normalized from peaks of 102 mm/h and 68 mg/L, hematuria resolved, and proteinuria decreased. Quantitative MPO‐ANCA, elevated at presentation, showed a progressive downtrend but remained detectable at last follow‐up. The renal course is best characterized as stabilization with partial improvement on a background of advanced chronic damage.

**Conclusion:**

This case highlights *M. abscessus* as a rare trigger of infection‐associated MPO‐ANCA glomerulonephritis and demonstrates that antimicrobial therapy combined with corticosteroids, without cytotoxic agents, can achieve renal stabilization and partial improvement. A downtrending MPO‐ANCA titer, rather than a static one, is the serologic pattern most consistent with a resolving infection‐associated process. Recognition of infection‐driven ANCA disease is essential to avoid potentially harmful cytotoxic therapy during active infection.

## 1. Introduction

Nontuberculous mycobacterial (NTM) infections are increasingly recognized as triggers of ANCA positivity and ANCA‐associated vasculitis (AAV), which can manifest as rapidly progressive glomerulonephritis. In a retrospective cohort of pulmonary NTM disease, Oda et al. found that 7 of 63 tested patients (11.1%) were ANCA‐positive, predominantly MPO‐ANCA, and 5 of these 7 (71.4%) developed AAV, most with glomerulonephritis [1].

Distinguishing infection‐associated ANCA disease from primary AAV is clinically crucial, as the former may respond to antimicrobial therapy and corticosteroids without cytotoxic immunosuppression (cyclophosphamide or rituximab). In infection‐associated MPO‐ANCA disease, the reported median interval from infection to vasculitis is ∼3 months, and ANCA titers characteristically decline once the infection is controlled [2]. Previously reported *Mycobacterium abscessus*–associated ANCA vasculitis described systemic microscopic polyangiitis requiring immunosuppression with corticosteroids, cyclophosphamide, and rituximab, complicated by recurrent vasculitis flares during treatment tapering and worsening NTM pulmonary disease with treatment escalation (Table 1) [3]. In contrast, to our knowledge, this is among the first reports of *M. abscessus* infection–associated MPO‐ANCA glomerulonephritis with partial renal improvement, resolution of hematuria, reduction in proteinuria, and a downtrending MPO‐ANCA titer, using corticosteroids and antimicrobial therapy without cytotoxic agents.

**TABLE 1:** Published cases of ANCA‐associated vasculitis triggered by nontuberculous mycobacterial infections.

Study	NTM species	ANCA type	Renal manifestations Biopsy finding	Immunosuppression	Antimicrobial therapy	Patient outcome
Current Case (2025/2026)	*M. abscessus* complex (MDR)	MPO‐ANCA	*M. abscessus* complex (MDR) MPO‐ANCA 70% globally sclerosed glomeruli; necrotizing and crescentic GN	Corticosteroids only (IV methylprednisolone 500 mg × 3 days, then oral prednisone 1 mg/kg)	Imipenem, omadacycline, clofazimine, inhaled amikacin	Improvement of hematuria and proteinuria; stable renal function
Addy et al. (2018) [3]	*M. abscessus*	MPO‐ANCA	*Microscopic polyangiitis* with neuropathy	Corticosteroids + cyclophosphamide, later rituximab	Prolonged antibiotics	Difficult disease control; vasculitis flares with immunosuppression
Asano et al. (2016) [4]	*M. avium* complex	MPO‐ANCA	Crescentic GN with immunodeposition (AAV + IRGN)	Corticosteroids	Antibiotics for MAC	Improvement in AAV, IRGN, and lung findings

## 2. Case Presentation

A 57‐year‐old woman with a 4‐month history of chronic cough managed by an outside pulmonologist presented with acute kidney injury. Evaluation revealed acute kidney injury with proteinuria and microscopic hematuria with dysmorphic red blood cells, consistent with a glomerular source [5].

Serologic workup was notable for MPO‐ANCA positivity, while PR3‐ANCA, complement (C3 and C4), anti‐GBM antibody, anti‐dsDNA, ANA, and infectious serologies (hepatitis B/C, HIV, syphilis, and coccidioidomycosis) were negative. Kidney biopsy showed pauci‐immune necrotizing and crescentic glomerulonephritis (Figure 1) with ∼70% globally sclerosed glomeruli (Figure 2), consistent with ANCA‐associated glomerulonephritis with marked chronicity. The absence of significant glomerular inflammation on immunofluorescence refers to the absence of immune‐complex deposition and endocapillary proliferation, consistent with a pauci‐immune process (Figure 1). Chest CT revealed localized cystic densities and ground‐glass opacities.

**FIGURE 1:**
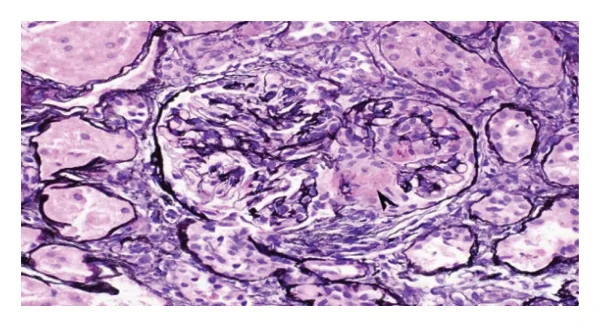
A viable glomerulus shows segmental necrosis with rupture of capillary loops and accumulation of fibrin (arrow). (Jones silver, 400*×* magnification). No significant glomerular inflammation was present, and IF and EM examinations were negative for immune deposits.

**FIGURE 2:**
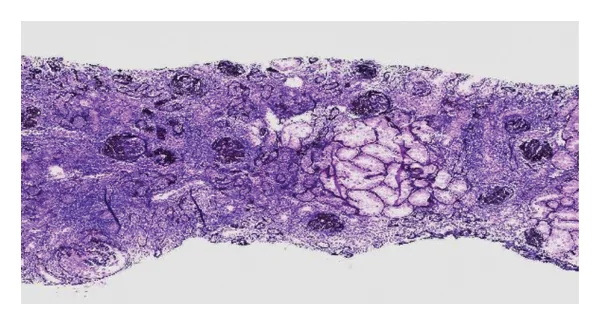
The biopsy showed significant chronic injury with approximately 70% globally sclerosed glomeruli, marked tubular atrophy, and dense tubulointerstitial inflammation (Jones silver, 100*×* magnification). Teaching point. In a patient with MPO ANCA positivity and rapidly progressive glomerulonephritis, always actively exclude chronic infection before labeling the disease as primary ANCA‐associated vasculitis and starting cytotoxic therapy. Infections such as nontuberculous mycobacteria can drive a secondary, reversible ANCA‐mediated process. Treating the infection and using limited corticosteroids can stabilize kidney function and improve hematuria and proteinuria. Premature use of cyclophosphamide or rituximab in active infection can worsen outcomes.

Following the institutional preimmunosuppression protocol, QuantiFERON testing and AFB cultures were obtained; because active mycobacterial infection had to be excluded before cyclophosphamide or rituximab, these agents were withheld pending culture results. Bronchoscopy with bronchoalveolar lavage grew *M. abscessus* complex, with susceptibility testing demonstrating multidrug resistance.

### 2.1. Clinical Timeline

Serial laboratory values are summarized in Table 2.

• Day 0 (presentation): AKI was identified; serum creatinine was 2.7 mg/dL 10 days before presentation (preillness baseline unknown), rising to 3.2 mg/dL at presentation. MPO‐ANCA was positive. Hematuria and proteinuria were present.
• Days 1–7: Serum creatinine reached a peak of 3.9 mg/dL. ESR and CRP peak levels were at 102 mm/h and CRP 68 mg/L, respectively. A kidney biopsy was performed. The patient was started on pulse IV methylprednisolone 500 mg daily × 3 days and then oral prednisone 1 mg/kg daily with a subsequent taper. Immunosuppression beyond corticosteroids was withheld pending AFB cultures. Creatinine slightly improved and remained between 3.2 and 3.4
• Weeks 2–4: BAL culture returned *M. abscessus* complex. Multidrug antimicrobial therapy was initiated per infectious diseases. Serum creatinine improved to ∼2.2 mg/dL.
• Months 2–6: Renal function further improved to ∼1.9 mg/dL and stabilized. ESR and CRP normalized. Hematuria resolved; proteinuria decreased. Quantitative MPO‐ANCA showed a downtrend but remained detectable.
• Follow‐up interval: The follow‐up period was approximately 6 months from presentation to the last recorded assessment.

**TABLE 2:** Longitudinal lab data from EMR.

Parameter	Baseline (Day 0)	Peak	Final (∼6 months)	Reference range
Serum creatinine (mg/dL)	2.7–3.2	3.9 (peak renal function)	1.9	0.6–1.1
eGFR (mL/min/1.73 m^2^)	∼19–16	∼13	∼29	> 90
UPCR (mg/g)	500	620 (peak)	180	< 150
UACR (mg/g)	270	340 (peak)	90	< 30
ESR (mm/h)	88	102 (peak)	14	0–20
CRP (mg/L)	55	68 (peak)	3	< 5
*Microscopic hematuria* (RBC/hpf)	30 (dysmorphic)	40 (peak)	0–2 (resolved)	0–2
MPO‐ANCA (AU/mL)	78	96 (peak)	34 (downtrending, still positive)	< 20

### 2.2. Treatment and Outcome

The patient received pulse intravenous methylprednisolone 500 mg daily for 3 days followed by oral prednisone 1 mg/kg daily with a subsequent taper. Given the active *M. abscessus* infection, cyclophosphamide and rituximab were withheld. Antimicrobial therapy for the multidrug‐resistant *M. abscessus* isolate was directed by susceptibility testing and designed in collaboration with infectious diseases. The isolate demonstrated no inducible (erm(41)‐mediated) or mutational macrolide resistance; accordingly, a macrolide‐containing multidrug regimen was used, consistent with the 2020 ATS/ERS/ESCMID/IDSA NTM guideline recommendation of a macrolide‐containing regimen with at least three active drugs in the initial phase for macrolide‐susceptible disease [6]. The regimen comprised oral azithromycin 250 mg once a day, intravenous imipenem 500 mg every 24 h with a renally adjusted dose, oral omadacycline 300 mg daily, clofazimine 100 mg daily, and inhaled amikacin 590 mg daily.

Over ∼6 months, serum creatinine improved from a peak of 3.9 mg/dL to 2.2 mg/dL and then 1.9 mg/dL, ESR and CRP normalized, hematuria resolved, and proteinuria decreased (Table 2). No cytotoxic immunosuppression was administered.

## 3. Discussion

This case illustrates infection‐associated MPO‐ANCA glomerulonephritis triggered by *M. abscessus*. Renal function stabilized and partially improved after antimicrobial therapy and corticosteroids, without cytotoxic agents.

The renal response is best described as stabilization with partial improvement on a background of advanced chronic damage, rather than complete resolution. Serum creatinine fell from a peak of 3.9 mg/dL to 1.9 mg/dL. Hematuria also resolved, proteinuria fell, inflammatory markers normalized, and the MPO‐ANCA titer trended down. Together, these findings support a meaningful response to corticosteroids and antimicrobial therapy. The size of the recovery still needs cautious interpretation, given ∼70% global glomerulosclerosis and residual CKD. Other possible contributors are resolution of a superimposed acute or prerenal component, the expected fall in creatinine after supportive care and corticosteroids, and the day‐to‐day variability of creatinine in advanced CKD. In ANCA‐associated glomerulonephritis, higher chronicity scores and higher presenting creatinine independently predict poorer renal recovery, so a complete recovery attributable to vasculitis would not be expected here [7, 8]. Most of the early creatinine improvement came after pulse corticosteroids, around the time antimicrobials were started. The relative contributions of corticosteroids, antimicrobial therapy, and resolution of a reversible AKI component therefore cannot be fully separated in a single case.

The serologic course is more informative than a single time point. In a systematic review of 23 patients with infection‐induced MPO‐ANCA vasculitis, Kakoullis et al. reported a median infection‐to‐vasculitis interval of 3 months. Vasculitis regressed after the infection resolved in 12 of 23 patients (52.2%). ANCA titers fell significantly on follow‐up in 14 of the 16 patients in whom they were measured and in all surviving patients with measured titers [2]. Van Gool et al. reviewed 182 cases of ANCA‐positive infective endocarditis, most with c‐ANCA/PR3‐ANCA (79%). All patients received antibiotics, and 39% also received immunosuppressants. During follow‐up, 69% became ANCA‐negative, and none was diagnosed with systemic vasculitis [9]. Because that cohort was mainly PR3‐ANCA with endocarditis, extrapolation to MPO‐ANCA after NTM infection is limited. A static or persistently high titer would therefore argue against a resolving infection‐associated process and raise concern for smoldering primary disease. In this patient, the quantitative MPO‐ANCA titer rose early in the course and has since trended down steadily with infection control and corticosteroids, in parallel with clinical and biochemical improvement. A falling titer, rather than static positivity, is the pattern consistent with a resolving infection‐associated process. However, a falling titer is not diagnostic on its own. Titers also fall with treatment of primary AAV, and the correlation between ANCA titer and disease activity is inconsistent [10]. The titer has not yet normalized, and serial monitoring remains necessary. In primary AAV in remission, a rise in MPO‐ANCA has been associated with clinical deterioration (positive predictive value 79% in GPA/MPA) [11], and reappearance of MPO‐ANCA has been associated with relapse [12]. These data come from primary AAV, but they suggest that a later rise in titer in this patient should prompt reassessment.

Distinguishing primary AAV from infection‐associated disease matters because cytotoxic immunosuppression during inadequately treated infection may have catastrophic consequences [2]. However, infection‐associated ANCA disease does not always respond to infection control and corticosteroids. Addy et al. described *M. abscessus*–associated microscopic polyangiitis in which reducing immunosuppression triggered vasculitis flares, one causing retinal vein occlusion that required rituximab, while intensifying immunosuppression worsened the NTM disease (Table 1) [3]. Yoshii et al. reported NTM‐associated MPA that required glucocorticoids, rituximab, and plasmapheresis to achieve remission [13]. These reports show that antimicrobials plus corticosteroids alone may be insufficient in a subset of patients, and they call for close monitoring for progression [3, 13].

Proposed mechanisms of infection‐associated ANCA production include release of neutrophil extracellular traps containing MPO and PR3 and stimulation of B cells by bacterial unmethylated oligodeoxynucleotides through Toll‐like receptor 9 [14]. Loss of immune tolerance and molecular mimicry have also been proposed, but these remain incompletely defined for MPO‐ANCA. Kidney biopsy showing pauci‐immune glomerulonephritis remains essential for diagnosis.

No follow‐up biopsy was performed, so resolution of the active necrotizing and crescentic lesions (Figure 1) cannot be demonstrated. It can only be inferred from the clinical and serologic course and remains unproven. The resolution of hematuria supports this inference because histologic activity is unlikely without hematuria. Kidney recovery can also occur despite advanced chronic damage [15]. Although peak creatinine (3.9 mg/dL) exceeded the KDIGO threshold for considering plasma exchange, it was not used given the modest expected absolute benefit at this level of kidney function, extensive chronic damage (Figure 2), the absence of dialysis requirement or alveolar hemorrhage, and the increased risk of serious infection during active NTM disease [15]. The outcome is best described as stabilization with partial improvement on a background of advanced chronic damage, with residual CKD (creatinine ∼1.9 mg/dL) and an MPO‐ANCA titer that is falling but still positive.

## 4. Conclusion

This case demonstrates infection‐associated MPO‐ANCA glomerulonephritis secondary to *M. abscessus*, with renal stabilization and partial improvement achieved using antimicrobial therapy and corticosteroids without cytotoxic agents. A downtrending MPO‐ANCA titer paralleling infection control, together with resolution of hematuria and reduction in proteinuria, supports an infection‐associated rather than primary process, though the titer has not normalized and the modest creatinine improvement may partly reflect resolution of a reversible AKI component superimposed on advanced CKD [2, 8]. When ANCA positivity occurs during active NTM infection, careful evaluation and serial quantitative ANCA monitoring are essential [3, 13].

## Funding

No funding was received for this manuscript.

## Consent

The authors declare that they have obtained informed consent from the patient discussed in the report for publication of this case, including relevant clinical information. Every effort was made to protect patient identity and confidentiality.

## Conflicts of Interest

The authors declare no conflicts of interest.

## Data Availability

The data that support the findings of this study are available from the corresponding author upon reasonable request.
